# Steps toward improving ethical evaluation in health technology assessment: a proposed framework

**DOI:** 10.1186/s12910-016-0118-0

**Published:** 2016-06-06

**Authors:** Nazila Assasi, Jean-Eric Tarride, Daria O’Reilly, Lisa Schwartz

**Affiliations:** Department of Clinical Epidemiology & Biostatistics, McMaster University, Hamilton, ON Canada; Programs for Assessment of Technology in Health (PATH), St Joseph’s Healthcare, Hamilton, ON Canada; Centre for Health Economics and Policy Analysis, McMaster University, Hamilton, ON Canada

**Keywords:** Ethics, Health technology assessment, Framework, Model, Tools

## Abstract

**Background:**

While evaluation of ethical aspects in health technology assessment (HTA) has gained much attention during the past years, the integration of ethics in HTA practice still presents many challenges. In response to the increasing demand for expansion of health technology assessment (HTA) methodology to include ethical issues more systematically, this article reports on a multi-stage study that aimed at construction of a framework for improving the integration of ethics in HTA.

**Methods:**

The framework was developed through the following phases: 1) a systematic review and content analysis of guidance documents for ethics in HTA; 2) identification of factors influencing the integration of ethical considerations in HTA; 3) preparation of an action-oriented framework based on the key elements of the existing guidance documents and identified barriers to and facilitators of their implementation; and 4) expert consultation and revision of the framework.

**Results:**

The proposed framework consists of three main components: an algorithmic flowchart, which exhibits the different steps of an ethical inquiry throughout the HTA process, including: defining the objectives and scope of the evaluation, stakeholder analysis, assessing organizational capacity, framing ethical evaluation questions, ethical analysis, deliberation, and knowledge translation; a stepwise guide, which focuses on the task objectives and potential questions that are required to be addressed at each step; and a list of some commonly recommended or used tools to help facilitate the evaluation process.

**Conclusions:**

The proposed framework can be used to support and promote good practice in integration of ethics into HTA. However, further validation of the framework through case studies and expert consultation is required to establish its utility for HTA practice.

## Background

There has been an increasing awareness of the need to incorporate ethics into the health technology assessment (HTA) process. This need is a consequence of the recognition that addressing moral and ethical issues can increase transparency and accountability of the HTA process and lead to better informed healthcare decisions [1]. As a result, HTA producers and decision-makers are increasingly more interested in considering contextual normative issues and value judgments, in addition to the results of clinical and economic evaluations in HTA.

Despite its importance, integration of ethical aspects into HTA remains challenging for several reasons. One of the key challenges is the plurality of ethical methods that need to be understood by HTA professionals in order to be applied appropriately [2, 3]. Our systematic review of existing guidance documents for ethical analysis in HTA suggested that methods proposed to address ethical issues differ considerably in terms of philosophical approach, structure, and comprehensiveness, and that there is no “one right way” to evaluate ethical considerations around healthcare technologies [4]. Another challenge is that HTA agencies too often fail to adopt the existing ethical guidance documents because most of the guidelines tend towards complexity and call for expertise, time and other resources that might not exist in their organizations. A recent survey of international HTA agencies revealed that only in 15 % of the participating agencies, ethical evaluations were typically performed by individual, mainly external, ethicists or multi-disciplinary teams including ethicists. The majority of the surveyed HTA organization relied on non-ethicist HTA professionals to conduct ethical evaluations, when required [2].

We believe if HTA professionals are expected to take part in ethical evaluations, they need a systematic approach that places greater emphasis on the process and steps required for merging ethics review activities with traditional HTA. This systematic approach should help HTA practitioners better understand the ethical evaluation process, use relevant ethics tools and seek appropriate expert guidance in answering ethical questions, if needed.

A number of existing guidance documents come closest to fulfilling this need. However, they rarely focus on the operationalization of their proposed approaches. For example, the ethical evaluation component of the European network for HTA (EUnetHTA)’s Core model [5], is a comprehensive document that provides a checklist of questions covering ethical issues related to the technology and the HTA process, describes commonly used methods to answer the questions, and proposes a standardized reporting structure. Likewise, the guidelines developed by a number of European HTA agencies, including the Austrian [6], Danish [7], French [8], German [9], Norwegian [10], Spanish [11], and Swedish [12] agencies, promote similar systematic approaches to integrating ethics into the HTA process. However, all of these guidance documents provide few details on what is needed to be done in order to implement the proposed methodology in a routine HTA environment. A further step has more recently been taken by the Swedish Council on HTA to provide a framework that takes into account not only the nature of the HTA process, but also organizational, financial and regulatory elements, as well as the availability of ethical expertise [13]. This framework that focuses on the identification and prioritization of ethical considerations in HTA provides only a brief description for operationalizing the ethics review process.

For HTA researchers with limited experience of performing ethical evaluations, there is still a need for a procedural guidance which would enhance their understanding of how an ethics review can take place during a HTA process and to aid them in incorporating ethical evaluation steps into a typical HTA plan. The aim of this paper is to fill this gap by offering a structured action-oriented framework and a list of literature-driven supporting tools. It should be noted that we do not intend to “reinvent the wheel” by proposing an alternative approach to substitute existing ethical inquiry methods. Rather, our study is intended to provide a process-based framework that encompasses a range of evaluative actions provided by other ethical guidelines for HTA in order to illustrate and describe the steps that are expected to be taken by a HTA team in evaluation of ethical considerations. We believe such a framework would allow HTA producers not only to understand the ethics review process, but also to make use of ethics in their assessments, by identifying interconnections and overlaps between ethics and other domains of HTA. Our framework also brings together the procedural steps and potentially helpful tools to provide more flexibility to the ethical evaluation process in HTA and increase its applicability.

The remainder of this paper is organized as follows: we begin with a description of our multiphase research methodology. Then, we introduce our stepwise framework and explain some important considerations which should be taken into account at each particular step. Next, we introduce a number of the most commonly used tools in ethical evaluation. Finally we offer further discussion of our proposed framework and draw conclusions.

## Methods

We initially performed a systematic review of the literature, published up to October 2013, with the purpose of identifying and mapping existing frameworks for ethics in HTA and methodological guidelines from national and international HTA agencies. The review identified 21 methodological articles and 22 HTA guidelines of varying complexity and scope. Data was abstracted, through content analysis, on methodological features of the identified guidance documents, particularly their areas of focus, theoretical foundation, analytical approaches, supporting tools, and required expertise. More details about this phase of research have been published elsewhere [4].

Then, to identify factors that are likely to influence the ethics review process, we conducted a comprehensive search in the literature to create a list of main barriers and facilitators of ethical evaluation in HTA. In addition, we performed a survey of the main HTA producing agencies throughout the world to learn about their experiences, methodological preferences, and their perceptions of the key barriers and enablers to incorporation of ethics in HTA. Ethics approval for the survey was granted by the Hamilton Integrated Research Ethics Board at McMaster University (REB 13-103). Informed consent was obtained using a recruitment email which described the aims of the study and notified participants that completion of the survey implied consent to participate in the survey. Therefore, a separate informed consent form was not required. The findings of the literature review and the survey are published in detail elsewhere [2].

The next stage was to draft a framework based on operational features of the identified guidance documents, as well as practical barriers to avoid and enablers to encourage in performing an ethical evaluation. We used data from the guidance documents included in our systematic review [4] to generate a comprehensive list of main elements and sub-elements that needed to be considered in an ethical evaluation and identify the common elements. The following a priori categories were utilized to group action items: scoping, data collection, analysis, and knowledge translation. However, as the analysis progressed, some preliminary categories were combined and further categories were added. The identified barriers and facilitators were used for further defining and customizing action categories and their related task statements [2]. The process was continued until seven final action categories were established: defining the objectives and scope of the evaluation, identifying stakeholders, assessing organizational capacity, framing ethical evaluation questions, ethical analysis, deliberation, and knowledge exchange or translation.

We generated a separate list of commonly recommended tools, which were identified in our systematic review [4], and examined all in relation to their application and appropriateness for each procedural step. Additional ethical tools were identified through targeted searches in the literature and consultation with subject matter experts. Finally, a summary table consisting of a brief description of the identified tools as well as some of the strengths and weaknesses of each tool was generated.

## Results

Our proposed framework consists of an algorithmic flowchart that illustrates a set of steps for operationalizing ethical evaluation throughout various stages of the HTA process; and a stepwise guide, which breaks down each step into individual task objectives and suggests some questions that need to be addressed at each step in order to complete the suggested tasks. To help facilitate the proposed evaluation tasks, a list of some commonly recommended or used tools is also provided, along with a brief description of their strengths and weaknesses. The Framework components are summarized below.

### The algorithmic flowchart

**Fig. 1:**
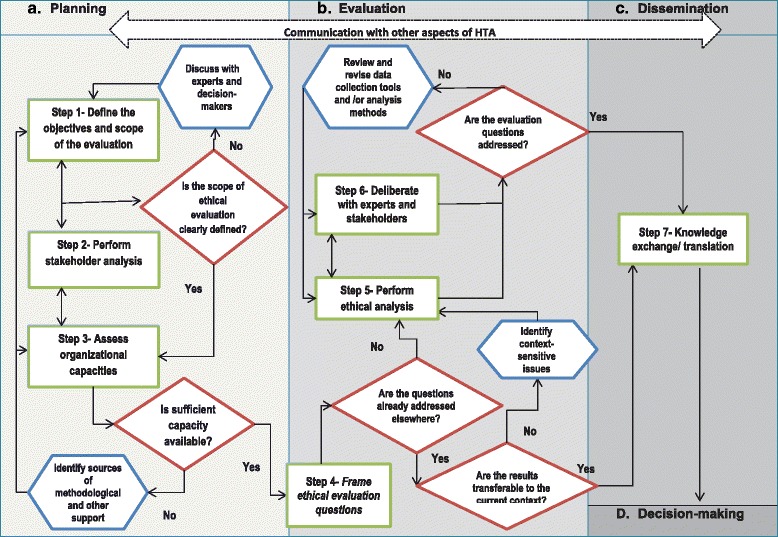
Algorithmic flowchart for ethical evaluation in HTA

As illustrated in Fig. 1, our stepwise flowchart organizes the actions required for an ethical evaluation practice into seven main steps and four conditional steps that allow for revisions and improvements within and across the main steps of the evaluation process. Although in this figure, progression from one step to another is shown to be linear, it is important to note that in real practice ethical evaluation activities can often occur simultaneously or iteratively.

### The stepwise guide

To make the framework useful for HTA practitioners, we have attempted to operationalize it into a guide, which can be seen in Table 1. The guide includes a non-exhaustive list of tasks and related questions which are drawn from our systematic review of ethical guidelines for HTA [4], input from experts in the fields of HTA and ethics (*n* = 6), and from our study of barriers and facilitators of ethical evaluation in HTA [2]. As with many ethics frameworks in other contexts, this guide is intended to aid HTA practitioners in identifying key steps that should not be overlooked, while also helping them to think about possible action items.

**Table 1:** Stepwise guide for the ethical evaluation process in HTA

Steps	Evaluative tasks	Potential questions
*1) Defining the objectives and scope of the evaluation*	□ Clarify the objectives and the scope of the HTA project	- What is the purpose of the HTA project (e.g., providing input for decision-making, formulating recommendations for practice guidelines, serving academic purposes)? - What is the rationale for the assessment of the technology (e.g., changing current practice, uncertainty/disagreement about benefits or risks of the technology)? - What are the information needs of potential users of the HTA findings?
	□ Consider ethical issues around the HTA project itself	- Why is the assessment undertaken? Who has requested it? - Is there any special interest in the assessment or pressure from authorities, manufacturers, patient groups, etc.? - Is there any conflict-of-interest concerns?
	□ Identify existing knowledge base about the technology	- Are there any characteristics of the technology that may raise ethical concerns (e.g., risk/benefit profile, utilization in vulnerable populations, access issues, modes of application) - What is the current practice? - What is the desirability of the technology (e.g., positive or negative utility values, QALYs) - What are the costs and organizational requirements for the implementation of the technology?
	□ Specify the objectives of ethical evaluation	- What the HTA team/organization intends to achieve by performing and ethical analysis (e.g., a description of ethical issues around the technology, identifying and resolving uncertainties around implementation of the technology by learning about stakeholder values and societal interests or through philosophical reflection)?
*2) Identifying stakeholders*	□ Identify potential stakeholders; engage key stakeholders to identify other stakeholders	- Who (potential groups or individuals) might affect or be affected (benefit/loose) by the introduction of the technology (e.g., decision-makers, manufacturers, healthcare providers, societal actors, patients and their families)?
	□ Identify the ways in which the above groups may be affected by the implementation of the technology	- What are the potential consequences of implementing the technology on disadvantage groups (access, equity, etc.)? - What are the potential consequences of implementing the technology on other stakeholders?
	□ Identify the ways in which the above groups may affect the implementation of the technology	- What are the known interests of stakeholders in the implementation of technology? - What opportunities (level of power) do stakeholders have to get involved in making decision about the implementation of the technology?
*3) Assessing organizational capacity*	□ Define key requirements	- What are the policy directions and priorities of the HTA organization and how might these influence evaluation of ethical considerations? - Is there a shared understanding of objectives and outcomes of HTA and ethical evaluation? - Are the opinion leaders in the organization supportive of integrating ethics in HTA? - Do the project timelines allow enough time for the completion of an ethical evaluation? - Are there any feasibility issues regarding ethicist involvement or stakeholder engagement? - Does the organization have any previous experience with ethical evaluations?
	□ Establish a team consisting of ethical expertise, HTA practitioners with experience in evaluation of normative aspects of healthcare technologies, and relevant stakeholders (when needed)	- Is the ethical expertise available in house? If not, are any external ethicists available to be recruited for the purpose of this evaluation? - Are there sufficient staff members with required characteristics (knowledge, skills and attitude) available to take part in the ethical evaluation?
*4) Framing ethical evaluation questions*	□ Recognize potentially relevant ethical problems and solutions that may arise from the introduction of the technology	- Is there any potential conflict between the technology and basic human rights, social and cultural values, patient’s autonomy, etc.? - What are the moral characteristics of the technology (e.g., risk/benefit profile, health improvement at the individual and society levels)? - Does implementation the technology require any life style modifications? - What are the long term effects of the technology on the users, their family members, and society (e.g. psychological impact, discrimination)?
	□ Map the current practice from an ethical perspective	- What are the key problems with the current use of technology (e.g., costs, equity problems, privacy, misuse of technology, freedom)? - What are the affected groups’ perceptions about the current practice?
	□ Identify sets of governance steps that might be necessary to resolve potentially relevant issues	- What solutions have been proposed to deal with the identified ethical problems? - How effective these solutions have been reported to be?
	□ List ethical issues around the technology	- Have I been able to identify any ethical issues around the technology (e.g., outcomes of medical choices, society’s access to the technology, ethically controversial situations at political or local levels, and ethically challenging situations at societal or healthcare system levels)?
	□ Justify what issues should be included in the ethical analysis, and why	- Which of the identified ethical issues are more relevant to the HTA project’s goal, and why? - Which of the identified ethical issues are more important, and why?
	□ Use dialogues and/or other deliberative methods for input seeking from ethical and technical experts as well as potential users, if necessary	- Has the plausibility of the identified ethical issues been stablished or discussed? - What insights are available from experts or stakeholders to aid in finalizing ethical evaluation questions?
*5) Ethical analysis*	□ Choose an appropriate methodology to address identified ethical dilemmas	- What methodologies are described in the literature or have been employed by others to study similar problems? - What theoretical paradigm is chosen by the research team to inform the ethical evaluation (utilitarian theory, deontological ethics, virtue ethics, etc.)? - What is the most practical and reliable approach to collect and analyze ethical data, considering the purpose of analysis, available expertise and other resources and feasibility of stakeholder engagement (e.g., empirical approach [using quantitative data], philosophical approach [using ethical theories and principles], narrative approach [using facts, value judgments, and stakeholder preferences], or a mixed approach)?
	□ Justify the choice of method	- What theoretical paradigm best fits the evaluation questions? and why? - How the selected approach might be helpful in answering evaluation questions?
	□ Review existing information and acquire additional relevant information through: - An extensive search in quantitative and qualitative literature - Deliberative methods	- Have adequate data been collected to serve the purpose of the ethical analysis? - What information is available in the literature about ethical, social or legal impact of the technology? - Is there any (retrospective, current or futuristic) information available on the use of the technology in different social and cultural contexts? - What arguments are available in the literature in favour of or against the technology? - What are the stakeholders’ values and preferences? - What controversies and potential conflicts exist at the local, societal and political levels around implementation of the technology?
	□ Ensure data from all sources are considered for analysis	- Has data from all possible sources collected for the ethical analysis (e.g., quantitative and qualitative evidence, stakeholder hearings, and expert opinion)? - Is triangulation of data sources possible?
	□ Examine the collected data for logic and coherence, validity and reliability	- What is the level of internal consistency of data? Is the collected data reliable? - Is there any self-contradiction or incoherence in the collected data? Does a fundamental logic exist among the collected facts and values? - What are the e factors that could influence generalizability of the evaluation results?
	□ Synthesize and integrate collected data (facts and values) into ethical arguments - Apply the principles of biomedical ethics - Perform philosophical arguments on the ethical questions from the perspective of ethical theories - Reflect on possible solutions	- Does the implementation or use of the technology challenge the basic principles of biomedical (e.g., beneficence, non-maleficence, autonomy, justice, vulnerability,) - What are the key arguments in favour of using the technology? - What are the key arguments against implementation of the technology? - Are clinical or economic benefits of the technology justifiable from the chosen (various) ethical perspective(s)? - Are the arguments sound and clear? - What are the possible options for acting, and their consequences?
	□ Acknowledge your own values and philosophical interest	- What is your position (perspective) on the matter? - How would you interpret the data, if you were in the stakeholders’/policy-makers’ shoes? - How confident are you that your position will remain the same in the matter over time?
*6) Deliberation*	□ Discuss the results of evaluation with an expert group to assess their relevance and completeness	- What do the experts have to say about the relevance of the collected data? - Do the experts have any suggestions as to what other sources of relevant information are available?
	□ Choose an appropriate method to discuss the results of ethical analysis with relevant stakeholders to seek their feedback on the results	- Who are the appropriate stakeholders to take part in or provide feedback on the analysis? - What are alternative sources of values for interpreting ethical analysis findings? - What are the ways that encourage identified stakeholders to provide required information. - What are the main concerns, preference, and emergent needs of stakeholders? - To what extend the stakeholder engagement activities have captured required information?
	□ Seek additional expert insight, if necessary, to ensure about the plausibility of the produced results during stakeholder hearings.	- Is it required/worth to engage a group of experts in a discussion of the ethical evaluation results? - Do you have any specific questions/uncertainties which you would like the experts to address?
*7) Knowledge exchange/translation*	□ Refine your target audience that might be interested or may benefit from the results of HTA	- Who is the target audience (e.g., policymakers, healthcare providers, patient groups, academic audience)?
	□ Refine information needs of your target audience	- What are the ways in which the report will be used (e.g., direct use of knowledge for problem-solving, conceptual use of knowledge for perception-shifting or understanding, political use of knowledge for supporting or challenging policy decisions)?
	□ Structure a presentation format to address the information needs of target audience	- How should the evaluation results be made available to users (in terms of content and format)?
	□ Report the results of ethical analysis in a transparent and effective manner	- Are the criteria and logic for the choice of methodology and selection of stakeholders disclosed? - Are the identified gaps in the literature, concerning ethical issues and values, addressed? - Are all favorable and non-favorable arguments reported? - Are anticipated changes that may follow from the implementation of the technology discussed? - Are the findings summarized and the most important value issues highlighted?
	□ Integrate knowledge translation in all steps of the assessment	- Has there been an integrated flow of information among team members working on different aspects of the technology (clinical, economic, ethical, social, legal, and organizational aspects) throughout the HTA process?

#### Step1. Defining the objectives and scope of the evaluation

Before starting an ethical assessment, it should be ensured that the objectives of both the overall HTA, to which the ethical evaluation will be incorporated, is set. In doing so, the role of ethics should not be over- or under-estimated. Rather, the scope and aims of ethical evaluation should be proportional to the candidate technology and the context [14]. A careful consideration of potential ethical issues around the primary motivations for conducting an HTA is also required at the scoping step, such as special interests of certain stakeholders in the assessment or external pressure from authorities, manufacturers, and patient groups. Then, as with any other evaluation process, ethical assessment should begin with an exploratory phase to identify the existing knowledge base surrounding the technology of interest such as technological aspects, modes of application, range of possible clinical indications, safety issues, as well as the therapeutic, economic and organizational impacts of the technology. Identifying the purpose of the ethical evaluation is the next essential task, as it will feed into the type of research questions, study design, and choice of ethical analysis approaches.

#### Step2. Stakeholder analysis

Given the importance of stakeholder interests and values in an ethical evaluation and their influences over potential decisions, it is good practice to conduct a stakeholder analysis during the defining and scoping phase to make sure that values and preferences of potential stakeholders are effectively included in the ethical analysis. Nonetheless, stakeholder analysis can be undertaken throughout all steps of ethical evaluation.

During the scoping phase, potential stakeholders can be identified through brainstorming, collecting and analyzing quantitative or qualitative information, and asking identified stakeholders who they would suggest as relevant stakeholders for the technology of interest. A typical stakeholder analysis involves assessing the interests of the identified key stakeholders, such as patients, healthcare providers, decision makers, family members and the general public in the candidate technology; assessing their importance and level of influence over the HTA process and decision-making; determining who stands to benefit and who stands to lose if the candidate technology is introduced, in what ways and to which extent; and identifying the most appropriate ways to engage stakeholders [15, 16]. Some useful tools for stakeholder analysis are introduced in Table 2.

**Table 2:** Commonly used tools for ethical evaluation in HTA

Tool	Description	Strengths	Challenges	References
A. Ethics literature review and appraisal				
Methodologies for the search and retrieval of information on ethical issues in HTA	Methodological approaches for the systematic retrieval of ethical information are discussed in two articles. These articles provide recommendations for good practice in selection of sources of ethical information, designing and executing ethics-specific search strategies, quality check of search results, and reporting information retrieval process.	Encourages a separate literature search relevant to ethical questions, using the common retrieval framework for effectiveness assessments.	The proposed search terms or strategies might not be sufficient for retrieval of all relevant ethical issues. Additional targeted searches might be necessary.	[31, 32]
Tools for critical appraisal of empirical ethics research	An article by Strech discusses the appropriate criteria for appraisal of empirical research required for ethical reasoning. He suggests four appraisal criteria related to the relevance of study questions, selected outcomes and measure, study design and generalizability of study results.	Addresses some important challenges of considering empirical data in ethical analysis.	No detailed guidelines or case studies are provided for how to apply the appraisal criteria.	[33]
	Mertz et al propose a set of structured quality criteria which can be used as a checklist to guide empirical ethics researchers and appraisers in the following four domains: research methodology, scientific and social relevance of the research project, interdisciplinary research practice, and research ethics.	Designed based on an in-depth analysis of existing empirical ethics research and the opinion and experience of experts in the field of medical ethics.	The practicality of the criteria is not tested in real life empirical ethics research practice.	[34]
A tool for critical appraisal of normative medical ethics literature	McCullough et al offer a tool to help clinicians (particularly obstetrician/gynecologists) in critical appraisal of normative bioethics literature. The tool incudes four questions about the focus of the study, validity and soundness of the study results, as well as their implication and usefulness in clinical practice.	Designed based on the standards of critical appraisal of argument-based ethics and evidence-based medicine.	Judgment about the validity and quality of ethical analyses and arguments requires some level of knowledge about ethical reasoning. This might not be an easy task for the target audience of the tool, i.e., physicians.	[35]
Guidelines for systematic reviews of ethical evidence	Strech e t al propose a 7-step approach for systematic reviews of empirical bioethics literature, The stepwise process involves definition of review questions, development and execution of search strategies, assessment of relevance and quality of identified studies, and analysis and presentation of data.	Practical recommendations are provided for each step. The application of the proposed approach is illustrated with an example.	The proposed search algorithms are not definitive and might need some modifications depending on the context and review questions. Data analysis and presentation may require some level of knowledge and skills in synthesis of qualitative data.	[36]
	Strech and Sofaer also offer a methodology for systematic reviews of non-empirical reason-based bioethics literature. Their model provides instructions for formulation of review questions and study selection criteria, identifying eligible literature, data extraction and synthesis, as well as presentation of the review results.	Structured based on the common steps of a systematic review process. Provides a detailed description of operational steps, and examples of how to apply the model in practice.	Performing a “systematic” review based on this model might be time-consuming. This type of review requires some level of knowledge about ethical reasoning. be time-consuming and	[37]
B. Stakeholder analysis				
Stakeholder Power/Interest grid	This tool is a four quadrant matrix that classifies stakeholders in relation to the power that they hold and their level of interest in the technology. Power classification can be based on the ability of stakeholders to define or influence health care systems and services, change the way services are provided, or guide the public opinion.	Highlights the importance of actors and interest groups in the technology	The stakeholders interests, perceptions positions, and influence are subject to change	[38]
Stakeholder SWOT	A SWOT analysis (Strengths, Weaknesses, Opportunities, and Threats) can help in understanding the interests of key stakeholders, the actions they can take to support and the risks that they pose to implementation of the technology.	Can be used to stimulate and organize thoughts and discussions in stakeholder analysis.	Procedures for performing a SWOT-analysis are not clearly defined. The analysis is prone to subjective biases of the assessors.	[39]
C. Public/stakeholder engagement				
Exploring public values and preferences	A methodology document published by the National Coordination Centre for Health Technology Assessment (UK) presents the results of a systematic review of qualitative and quantitative approaches to involving the public in in HTA. The document identifies and describes details of the techniques that can be used to obtain public preferences and makes recommendations regarding the use of different techniques. Some of the commonly used methods identified in this document are as follows: − Quantitative techniques, including ranking (e.g., simple ranking, qualitative discriminant process, and conjoint analysis) rating (e.g., visual analogue scale) and choice-based (e.g., standard gamble, time-trade-off, discrete choice conjoint analysis and willingness to pay) methods. − Qualitative techniques, including individual interviews, focus group discussions, Delphi technique, citizen’s juries, consensus panels, and nominal group techniques.	Summarizes and compares various techniques in a single document. Uses pre-defined sets of criteria to evaluate methodological issues of different techniques (e.g., validity, reliability/reproducibility, generalizability, acceptability to respondents, or cost) identified methodologies. Provides examples of how the techniques have been used in research practice.	No single best technique or group of techniques for public engagement is recommended by this document. Users of the tool may require background knowledge and specific skills that enable them to choose and conduct an appropriate public engagement technique.	[40]
D. Identification and analysis of ethical issues				
The Socratic approach (Hofmann’s guiding questions)	This approach consists of 6 steps, whereof one step covers 7 main questions and 33 explanatory and guiding questions. This checklist is designed t for identification of and reflecting on ethical data throughout the HTA process, and for reflexive dialogue with stakeholders.	Takes into account several ethical perspectives and analytical approaches. Can be used by HTA practitioners who may be less familiar with ethical analysis. Facilitates ethical analysis.	Users of the tool may require some level of ethical knowledge in order to use appropriate approaches to answer the questions.	[41]
HTA core model’s assessment element cards (AECs)	AECs describe the details of the information that is outlined by the basic units of the HTA Core Model (assessment elements). Each AEC provides information on the element, its importance and transferability for different applications (diagnostic, surgical, pharmaceutical or screening technologies), and appropriate sources of information and research methodologies to address the question defined by the element. The ethical domain of the Core Model includes 19 elements related to the 19 ethical issues on the topics of beneficence/non maleficence (4 AECs), autonomy (4 AECs), respect for persons (3 AECs), justice and equity (3 AECs), legislation (2 AECs), and ethical consequences of HTA (3 AECs).	Designed to provide structured information required for answering the generic question defined by each assessment element. Useful when producing HTA reports based on the HTA Core Model.	The way in which AECs should be used as a part of the assessment is not fully addressed in the model.	[5]
Ethical matrix	Ethical matrix is an analytical tool to aid ethical analysis of technological options The matrix uses a tabular format to identify ethical impact of a particular technology on different stakeholders. The table lists a set of prima facie moral principles, typically the four Beauchamp and Childress’s moral principles (autonomy, beneficence, non-maleficence, and justice), along one axis and different stakeholder groups along the other axis. Relevant facts and values are usually listed in each cell of the ethical matrix. Ethical matrix can be used either to identify ethical considerations around the technology or to quantify and compare the impact of the technology on different principles using semi-quantitative scores (e.g., ranging from -2 to +2).	Facilitates ethical analysis by simplifying and structuring ethical discussion Raises awareness of a wide range of ethical concerns Helps researchers and decision-makers to avoid bias towards a specific moral principle. Can be used in both expert-led and participatory/deliberative ethical evaluation processes.	May become large, complex and difficult to manage, when too many moral principles are listed or diverse groups of stakeholders are identified.	[42]
Consequences table	A summary table of consequences of using and not using a particular healthcare technology is recommended in the HTA core model as an open framework for performing ethical analysis. This table summarizes key benefits and adverse impacts of implementing of the technology or otherwise on various stakeholder groups. A consequences table summarizing positive and negative impacts of the technology on all domains of HTA, along with references to the quality of their evidentiary sources, is also proposed as a part of the HTA core model’s reporting template.	Allows for highlighting key impacts of a particular technology on various domains of HTA. Can be used by decision-makers to compare anticipated ethical issues around alternative technologies in relation to other domains.	Cannot be used as a substitute for careful ethical reflection	[5]
E. Computerized support tools for aiding ethical analysis				
EthXpert	EthXpert is a computer program designed to help the user in summarizing and structuring ethical problems, describing potential inter-relations between the interests of different stakeholders, and analyzing the impact of alternative technologies on various stakeholders’ interests.	Does not focus on a specific audience or any specific contexts. Therefore, can be applied to ethical evaluation in HTA.	In some cases, the use of the these computer programs can be difficult and time consuming, especially when one needs to include all details about complex ethical problems, or too many different perspectives. The use of the software may require investment in resources.	[29]
ETHOS	Ethos is a computer program that provides a framework for organizing, storing and analyzing ethical information needed for problem solving or decision-making. The program allows for ethical analyses using different ethical theories and approaches.	Illustrates the flow of data collection and analysis in a map format. Enables the user to add or remove information through an iterative process.		[30]

#### Step3. Assessing organizational capacity

The following resources are necessary in performing an ethical evaluation: a person with a strong educational background and experience in applied ethical theory, sufficient financial resources and time for conducting the evaluation, and the capacity for training, if needed. Therefore, it is important to assess the level of organizational readiness along each of these dimensions in order to balance available resources with the requirements of ethical evaluation.

It must be ensured that sufficient knowledge, experience, and skills exist in the organization to collect ethics-related data and to perform a comprehensive ethical analysis for several reasons. Firstly, since ethical evaluation is an approach which deals with norms and values, conducting such an evaluation would not be possible without a reasonable amount of knowledge of ethical theories and principles. Secondly, because the scope of ethics literature is wide and can include theory as well as both quantitative and qualitative study results, HTA practitioners involved in ethical evaluations should be able to effectively appraise ethics literature and reflect on the collected information. Thirdly, a well-performed ethical analysis uses moral reasoning rather than merely describing facts and values. Hence, the rigour of ethical reasoning is usually dependent on how skillfully the analysis has been performed. Furthermore, the capacity in methodologies associated with ethical analysis through public discourse is often lacking in some HTA organizations [17]. Therefore, to get involved in public participatory processes, HTA professionals might need to acquire a range of new skills in different methods of public engagement before getting involved in such research activities [14, 18].

#### Step4. Framing ethical evaluation questions

Recognition of existing ethical dilemmas or the ones that are perceived likely to emerge after implementation of the technology (hypothetical dilemmas) is essential for the formulation of ethical questions that need to be answered. Identification of the existing ethical issues that could be resolved through the introduction of the technology should also be accounted for. Zydziunate et al. [19] systematically reviewed ethical dilemmas that might affect decision-making within health care systems and suggested that ethical dilemmas in healthcare might happen in institutional, local or national levels. The review listed the following terms that had been commonly used in defining or discussing ethical dilemmas: “continuing balancing” between health care needs and budgets, “result of resource allocation”, “gap between professional obligations and possibilities”, “ethically controversial situation”, “concern about interactions”, “outcome of medical choices”, “concern about society’s access to healthcare resources”, and “ethically difficult or ethically challenging situation”.

In practice, it might not always be necessary to make a comprehensive list of existing ethical conflicts or controversial issues through a systematic inquiry. However, it is important to discuss and specify which of the recognized ethical considerations and arguments are more relevant to the assessment and provide a justification for why these could be relevant. One familiar example is the dilemma that might arise in situations involving genetic testing technologies. Genetic test results, by nature, can reveal aspects of the tested individual’s susceptibility to health problems (potential for stigmatization or discrimination). In addition, they may have implications on the blood relatives of the tested individual, who might also request to know their family member’s genetic test results (potential for information abuse or intrusion of privacy). These aspects of the technology should be explored and discussed while framing the ethical evaluation questions. At this phase of evaluation, a priority should be assigned to ethical questions that may have greater implications on decision making. However, the HTA team must allow flexibility for adding questions during the ethical evaluation process.

#### Step5. Ethical analysis

As it was previously mentioned, the present framework does not aim to provide instructions on how to perform an ethical analysis, but rather assumes that HTA team members who are responsible for the analysis of ethics data have the knowledge and skills to take on this important task. Ethics expertise can be critical at this step, depending on the type of the assessment. A normative (principle- or theory-based) evaluation should generally be performed with the help of experts with knowledge in ethical theory. In participatory or interactive assessments, where expert and lay opinions are considered equally valuable, ethicists can play an active role by providing rationale for potentially useful analytical approaches, scientific and theoretical inputs to stakeholder and public debates and assisting stakeholders in reaching a consensus [14]. In sensitive topics, it may be desirable to seek discussion from more than one ethicist. Other HTA practitioners (non-ethicists) can also have a role, although necessarily limited, in ethics review and analysis (e.g., helping with formulating ethical questions and searching for potential solution through systematically identifying and summarizing ethics-related data, helping with participatory research, etc.).

Our systematic review identified multiple guidance documents for the analysis of ethical data. The identified methodological approaches vary in their goal, philosophical approach, structure, and comprehensiveness. Traditional approaches consist of making ethical issues explicit by identifying facts and values, setting out arguments, providing reasons and justifying potential decisions through moral principles and theories (e.g., principlism, deontology, casuistry, and axiology). Deliberative approaches, on the other hand, advocate participatory and interactive approaches as complementary methods to traditional normative reasoning (e.g., wide reflective equilibrium, actor-network theory, interactive HTA, and social shaping of technology). It can be useful to employ more than one method in a given evaluation to help attend to the problem of bias, address the complexity of ethical dilemmas and uncertainties around healthcare technologies, and better justify HTA decisions [14, 20–23]. Different approaches can be compared and contrasted to more thorough and balanced results.

Hofmann et al. describe the most frequently used approaches for addressing ethics in HTA and provide guidance for choosing among various analytical approaches [24]. They believe that the selection of analytical approach for addressing ethical considerations in a specific HTA depends on the goal, context and process of HTA. Based on their classification of different methods of integrating ethics into HTA, the authors of this guidance document suggest that traditional bioethical approaches (e.g., deontology, casuistry, principlism, and axiology) are more suitable if ethical inquiry is performed as an independent assessment with less (subsidiary integration) or equal (combined integration) importance compared to other domains of HTA; while the approaches that are developed with a focus on the process of HTA (e.g., Socratic approach, interactive technology assessment, and social shaping of technology) are more appropriate when there are overlapping dynamics (coordinated integration) or continuous interactions (interactive integration) between ethics and other domains of HTA.

It is important to note that the results of ethical analyses may be influenced by the analysts’ knowledge, their experiences, values, and attitudes, as well as the technological, organizational, social, and political contexts in which the analyses are performed. Ethical analyses are also at risk of bias due to actual or potential conflict of interests in the HTA organization, such as which may influence the ways in which ethical information is collected, analyzed and interpreted. Therefore, it is good practice to provide a sound justification of the choice of the methods and disclose any financial and non-financial relationships with organizations or groups who may have an interest in the candidate technology and its implementation.

#### Step6. Deliberation

Once the preliminary analysis of ethical data is completed, it is desirable to discuss the results with the members of the multidisciplinary HTA team, and other experts if needed, in order to verify plausibility and reasonableness of the results. To ensure that the results are perceived as relevant by stakeholders and the public, it is also important to use public engagement methods to take in a variety of inputs from the groups whose values and preferences can provide a means for a better informed and legitimate policy decisions [25]. Although engagement of the public (including relevant stakeholders) in an ethical evaluation process, and in HTA in general, has been promoted at different levels, the common exercise is to gather the public input in an ad-hoc basis through deliberative methods such as surveys, focus group discussions, etc. Alternatively, the public input can be sought directly from the public representatives who are involved throughout the HTA process, or through an institutionalized approach, where the public or specific stakeholder groups are asked, as consultants, to provide input for decision-making in an ongoing basis [25]. Despite its importance, as a democratic exercise in ethical evaluation, public involvement methods can be challenging to perform due to their complexity, costliness, and time consuming nature [26].

Additional expert insight might be necessary to ensure the plausibility of the produced results during stakeholder hearings, before a particular conclusion is reached and the final report on ethical issues around the technology is written.

#### Step7. Knowledge exchange/translation

The purpose of HTA is primarily to support healthcare policy-makers in making evidence-informed decisions, and secondarily to help advance knowledge about a particular health technology and stimulate further research [27]. Therefore, the dissemination of the HTA findings, including ethical aspects, must be timely and appropriately tailored to the needs of potential users.

In order for the results of an ethical assessment to be utilized as an input for decision-making, the knowledge translation activities should begin in the earliest stages of the HTA process, through an effective interaction between HTA-producers and decision-makers, and continue throughout the evaluation [28]. The results need to be communicated in a manner that can be understood and easily utilized by decision-makers. The feedback from potential users should be received throughout the project and used to improve the quality of the research. HTA reports should address various dimensions of an existing or hypothetical ethical problem surrounding the technology of interest, using all relevant evidence from research and non-research sources, and applying suitable analytical approaches. It is also important to address how different stakeholders and members of society might be affected by the implementation of the technology or otherwise.

HTA findings can also be disseminated among other relevant target groups, such as healthcare researchers, clinicians, healthcare service providers (e.g. hospitals), third party payers, biomedical manufacturers, patients, and the general public. However, it is essential to translate the findings (including the results of the ethical analysis) into formats that are understandable and useful to the above-mentioned groups of audience [7].

### Selected tools

Table 2 provides a list of tools and techniques to support ethical evaluation tasks. This list does not include every possible tool that could potentially be used at each step of the proposed model, but has explored some of the more common ones that can assist HTA practitioners in evaluation of ethical considerations. It is important that HTA practitioners with responsibility for addressing ethical issues have a comprehensive knowledge and understanding of such tools in order to choose tools which are appropriate for varied types of ethical objectives and evaluation tasks.

Four categories of tools can be distinguished in Table 2: ethics literature review and appraisal, stakeholder analysis, exploring stakeholder values and preferences, and identification and analysis of ethical issues. We also searched for computerized support tools for aiding ethical analysis and identified a number of tools which were designed to help the users in summarizing and structuring ethical arguments, describing potential inter-relations between the interests of different stakeholders, or analyzing the impact of alternative technologies on various stakeholders’ interests. Although the selected computer programs, i.e., EthXpert [29] and Ethos [30], were originally offered as ethical decision-making support tools, they could potentially be applicable in ethical analysis for HTA.

## Discussion

We believe that, only by fully understanding all of the different steps of ethical evaluation and specific issues that may arise at each step, can HTA teams integrate ethics in their assessments. The framework presented in this article outlines the important steps that should be adopted by HTA practitioners for a comprehensive evaluation of ethical considerations. It is intended to guide HTA producers, especially those who are not accustomed to performing ethics reviews. The framework may also be used by researchers, evaluators, or decision-makers in order to critically appraise the process used for an ethical evaluation; or for educational purposes, especially to show the flow of activities needed for the evaluation of ethical considerations. Our framework may be a beneficial addition to the existing literature on ethics and HTA, for the following reasons:

Firstly, the framework is informed by the key operational features of existing ethical guidelines for HTA, as well as practical concerns and technical demands of potential user. It recognizes that ethical evaluations might be discounted or not undertaken in some HTA organizations because they are perceived as being impractical, resource consuming, or unfeasible. Therefore, the framework encourages proper scoping, strategic resource planning, and strengthening organizational capacity.

Secondly, the proposed framework aims to promote a more systematic and structured way of integrating ethics into HTA by mapping the relatively complex process of ethical evaluation and highlighting its main steps. Nonetheless, it is conceptually simple and employs terms and concepts that are familiar to the majority of HTA practitioners, including those who are less familiar with ethical evaluations. The visual representation of the stepwise process helps HTA practitioners to understand the nature of an ethical evaluation. The stepwise guide, on the other hand, reduces the complexity of evaluation by breaking down the procedural steps to smaller sets of tasks and providing guiding questions. With its focus on making the evaluation process more understandable and practical for all HTA practitioners, the framework can be easily used in training of HTA professionals, as well as also being able to play a role in harmonizing existing evaluation approaches across HTA organizations.

Thirdly, the stepwise framework accommodates the changing relationship between technology assessment, policy, and society by fostering the integration of stakeholder and public input into the ethical evaluation process. Furthermore, it promotes a holistic approach to evaluation and stresses that ethical issues need to be seen in interconnection with clinical, economic, social and legal issues, and that these relationships require an appropriate cooperation between the team members working in different domains of HTA. Another important aspect of the stepwise framework is its flexibility. The users of the framework are offered the possibility to choose the tasks that are more relevant to their assessments or to customize the evaluation process according to their needs.

In summary, the strengths of our proposed framework lie in its structured yet flexible approach to the evaluation of ethical considerations in HTA. It can, therefore, be helpful in building a more consistent practice of ethical evaluation among HTA professionals.

In spite of its strengths, our proposed framework also has limitations. Firstly, to use the framework HTA producers may require information, data and expertise and other resources that may not be readily available in a typical HTA organization. Qualitative evidence, stakeholder input and normative judgements are usually required to address ethics-related questions. Our framework fails to deeply address the ways in which ethical data should be tackled or specify how the evaluation process should be applied in different contexts. Secondly, the current version of our stepwise framework lacks an example case study where all the steps are applied. Furthermore, the validation of the proposed framework was not performed as part of this research project.

We will undertake further work to validate the framework and test its practicality through case studies, seeking stakeholder feedback, and expert opinion. Based on the results from the case studies and feedback received from the experts and potential users, we are planning to: (a) modify the stepwise framework by adding or removing steps; and (b) enhance its practicality by adding flow charts that illustrate details of various steps, and auxiliary tools or checklists to facilitate the ethical evaluation process; and (c) perform a final validation of the framework using further case studies.

## Conclusions

Despite the increasing attention given to the incorporation of ethical considerations in assessment of healthcare technologies, HTA producers continue to face challenges in integrating ethics to HTA. The intention of this research has been to construct a procedural framework that considers the nature and sequence of ethical evaluation process in the context of HTA. The proposed framework provides a conceptual foundation to allow for ethical issues to be addressed in HTA. Our framework can serve as a starting point towards a set of comprehensive strategic guidelines, as well as for the supporting and promoting good practice of integrating ethics in HTA. However, for a wider use and dissemination, its content needs to be applied in various HTA projects and validated through consultation with experts and policy makers.

## Abbreviations

EUnetHTA, European Network for Health Technology Assessment; HTA, Health Technology Assessment
